# An empirical study on the impact of genetics and gender and its consequences in hypertrophic cardiomyopathy

**DOI:** 10.21542/gcsp.2025.15

**Published:** 2025-02-28

**Authors:** Anshuman Gupta, Harneet Singh Khurana, Gurmeet Singh

**Affiliations:** Dayanand Medical College & Hospital Ludhiana, Punjab, India

## Abstract

Background: Gender-associated variations in phenotypic expression and their consequences are established in numerous cardiac circumstances. However, their impact is questionable in the case of HCM.

Objective: To investigate the demographic and clinical profiles of the HCM patients. Also, to compare the echocardiographic features according to the HCM subtypes in the study populace.

Methodology: The present study was conducted at the DMCH, Ludhiana, from March 2019 to May 2021, using a prospective observational and non-blinded design. The data regarding demographic and clinical profile are gathered for the specified duration. The clinical features are confirmed through the Echocardiography. The gathered data are analyzed through chi-square to determine the differences among the groups with the aid of the SPSS tool.

Results and discussion: The demographic profiles and clinical assessment of 103 patients are analyzed. The subjective assessment reveals that HCM is predominantly in males in a ratio of 2.1:1. Dyspnea is a chief complaint of both genders (77.67%). Apical type is prevalent in male HCM patients. MYBPC3 and MYH7 are the general mutations found in the genetic tests. SCD is found in patients possessing this type of genetic mutation. The non-obstructive type is more common than the obstructive type.

Conclusions: HCM is a chronic disease and causes morbidity as well as mortality globally. HCM patients are vulnerable to SCD and stroke. These risk factors rely on the diagnosis associated with age, gender, obstruction, obesity, and coronary diseases. Hence, the present research on the demographic characteristics of HCM patients promotes awareness regarding the complications among the Indian populace.

## 1. Introduction

### 1.1. Background of the study

Hypertrophic cardiomyopathy (HCM) is the most prevalent genetic cardiomyopathy and is present in diverse races and ethnic groups. Several individuals possessing HCM might lead a normal or near-normal life expectancy. Moreover, it is characterized by the marked variability in the natural history and morphological expression. On the basis of mutations, there will be a modification in the family transmission, hypertrophy degree, evolution, and forecasting of the disease^1^. The initial HCM description was proposed by Robert Teare in 1953 through the publication of a series of cases of muscular hematoma or asymmetrical hypertrophy among the young populace followed by sudden death. Subsequently, Dr. Eugene Braunwald demonstrated the histological aspects, clinical features, and treatment.

HCM is a genetically identified disease in the heart muscle caused by mutations in one of the numerous sarcomere genes that encode the constituents of the contractile apparatus. HCM diagnosis is based on the existence of LVH –non-dilated left ventricular hypertrophy^2^. It is detected by means of magnetic resonance imaging (MRI) and Echocardiography. It prevails in the absence of other systemic, cardiac, syndromic, or metabolic diseases. It is exhibited as asymmetric septal hypertrophy even though the other patterns, likely apical, lateral wall, and concentric, can exist. The epidemiological research reveals that the prevalence of one among five hundred populace is detected in 122 countries^3^. It has been reported that almost 20 million people are affected by the HCM globally.

HCM displays enormous diversity both phenotypically and clinically. The pattern, location, and extent of the LVH (left ventricular hypertrophy) are heterogeneous^4^. The abnormalities of HCM patients are LV outflow tract obstruction, Myocardial ischemia, systolic dysfunction, mitral regurgitation, and diastolic dysfunction. It generates a variety of symptoms encompassing dyspnea, fatigue, palpitations, chest pain, and syncope. HCM patients are more prone to supraventricular as well as ventricular arrhythmias and a high risk of sudden death^5^.

The etiology of the HCM is related to the gene-encoding proteins of the Z-disc, cardiac sarcomere, and calcium-controlling proteins^6^. 20 genes are associated with the disease, and almost 2000 dissimilar mutations have been detected in HCM patients. The genes involved in the HCM are MYH7 (myosin heavy chain) and MYBPC3 (myosin binding protein C). The genetic phenocopies are likely Danone’s disease, Fabry’s disease, amyloidosis, and Friederich’s ataxia^7^. The sarcomere mutations might also cause idiopathic restrictive cardiomyopathy, dilated cardiomyopathy, and non-compaction LV. HCM patients with the sarcomere mutations tend to have a two-fold risk higher compared to the other mutations.

Gender has proved to be significant in the characterization of acquired cardiac conditions. Since HCM is a genetic disease, gender differences are also a significant factor in evaluating the prevalence of HCM among the populace^9^. The gender modulated the occurrence, clinical course, and morpho-functional manifestations in the HCM. There will be modifications in the ion channel expression and remodelling of left ventricles among male and female HCM patients^10^. Clinical differences in gender arise due to complex environmental and societal discrepancies as well as biological variations^11^.

HCM is generally an inherited disease. Family history is a recording of the associations among family members with medical histories. It encompasses past and present illness and identifies the disease pattern in the family. Sudden Cardiac Death (SCD) is an unpredicted death caused by cardiac disease that prevails for a short duration. It generally occurs within one hour of the symptoms in an individual with a known or unknown cardiac ailment.

### 1.2. Significance of the study

The majority of the HCM patients have no or minor symptoms. Thus, the affected children and adults are often diagnosed with the family screening. The asymptomatic patients having minor symptoms are diagnosed better than the severe symptoms. The patients with milder symptoms will benefit from the prior diagnosis with the stable clinical course followed by effective treatment. The major complications of the HCM are ventricular arrhythmias causing chest pain, sudden death, and atrial arrhythmias encompassing embolic stroke^12^. The mortality of HCM patients is elevating from 1% to 4%. The risk factors are age, gender, obstruction, symptoms, arrhythmias, infective endocarditis, and heart failure. Therefore, the present study aids to analyse the impact of the gender and genetics and its consequences in the HCM.

### 1.3. Research objective

The objectives of the present research study are as follows:

1. To overview the clinical and demographic profile of hypertrophic cardiomyopathy in patients
2. To compare echocardiographic characteristics with respect to subtypes of hypertrophic cardiomyopathy in patients.

Section 2 describes the prevailing scholarly research works related to the present research. Section 3 provides the research methodology, and the analysis result is presented in Section 4. Section 5 illustrates the discussion as well as the limitations of the study. Lastly, Section 6 discusses the conclusion and future recommendations of the study.

## 2. Literature review

HCM is a heart muscle disorder and is inherited as an autosomal trait. The existing study^13^ identifies the epidemiological variables and fits a probability model which assumes the dominant mode of inheritance. 127 HCM patients are analyzed. The outcome of the analysis reveals that there has been a high male preponderance, and the sex ratio is found to be M: F 3.7:1. The age onset has been identified to be more than a decade in the familial case at 30 ± 10 years compared to non-familial cases at 44 ± 14 years. It brings out the complications of HCM and recommends a supplementary mode of inheritance apart from autosomal dominance. It also proposed that the mixed framework of inheritance is considered to be an optimal fit for complex disorders.

The prevailing study^8^ examines the clinical evaluation and epidemiological characteristics of HCM patients. It performs the analyses on the basis of gender, age, non-familial and familial status, and HCM subtypes. It contributes to the heterogeneity of conditions that make the prognosis much more challenging. The outcome of the analysis reveals that the sex ratio of 3.1:1 identifies the male preponderance. There are sporadic cases of 37% and the familial cases of 63%. The mean age is 38 years for the onset of HCM.

Additionally, it evaluates that the males were dominant at the age of 21 to 40 and exhibited the non-obstructive type. Alternatively, the female preponderance was identified in the age groups of 0–20 and 40–60. These groups have a high frequency of obstructive HCM related to severe manifestations. They also have poor prognosis on the basis of essential echocardiographic constructs.

The existing study^14^ aims to evaluate the clinical, demographic as well as genetic profile of HCM patients in India. The patients were selected on the basis of a WHO survey. Echocardiography is utilized to measure the clinical phenotypes, and the sequencing of the MYH7 gene was processed for both the control and experimental groups. 59 patients were clinically diagnosed, and there exists male preponderance in the ratio of 5.5:1. The mean age is 39.2 ± 14.5 years, encompassing familial HCM. It accounts for 18 percent of the total HCM families. The non-obstructive HCM type is more prevalent than the obstructive HCM. The posterior wall thickness of LV is 16 ± 4.8 mm. The familial patients have IVS thickness of 21 ± 8.3 mm. It has greater thickness compared to the sporadic patients. The research study detects three mutations among three Indian patients while sequencing MYH& hotspot.

The prevailing study^15^ insists that mutations are responsible for disease storage, which causes phenotypic resemblance to HCM. Routine testing and clinical identification of the family members are significant. It also reports that mutations of the dozen genes that encode the sarcomere-related protein cause HCM. MYBPC3 and MYH7 are the two chief genes involved and account for 50% of causing HCM in the families.

The existing study^16^ analyses the clinical characteristics, structure as well as functional variation, and prognosis of HCM associated with hypertension (HTN). Almost, 90 HCM patients with HTN and 172 patients without HTN are selected for examination. The two categorical groups were compared, and their clinical features, cardiac structure, and prognosis were analyzed. The outcome reveals that the HCM patients associated with the HTN had fewer syncope events in their medical histories (8% vs. 22%, *P* < 0.01). The SCD in the family is 3% vs. 10%, *P* < 0.05. The apical hypertrophy prevalence and obstruction in the mid ventricles is higher among the HTN group.

The prevailing study^17^ is to investigate the HCM patients from the cardiomyopathy group. It also analyses the demographic profiles and presentation mode on the basis of symptoms. It also categorizes the selected HCM populace into differential phenotypes on the basis of Echocardiography. 233 HCM patients are selected for the research analyses. The mean age was 53 ± 14.5years alm, ost 70% of male preponderance. 36% were asymptomatic, severely symptomatic (27%) and mildly symptomatic (37%). The patients were categorized into six phenotypes comprising five classic phenotypes and one atypical. The reverse curvature is prevalent (49%), and 19.3% are symptomatic phenotypes. The remaining 19% were apical, 6% sigmoid, 11% were neutral, and 4% were atypical. The ratio of male and female for the reverse curvature was 3:1, the apical phenotype was 2.4:1. Finally, the neutral phenotype was 2:1. The female-to-male ratio was 2.5:1 in the sigmoid phenotype. It concludes that a complete assessment is required for HCM patients. The symptom status relies on the age, LVOT obstruction, segment thickness, and septal contour.

The existing study^18^ investigates the prevalence and incidence of HCM in Korea. The reports are extracted from the *KoreanNationalHealthInsurance* database from 2010 to 2016. It has been reported that the HCM steadily increased from 0.016% to 0.031% from 2010 to 2016. This gradual increase was identified in both genders. The male predominance was identified. The ratio of male to female is 2:1, with no variation in the study period. The HCM prevalence elevated slowly in the age group of 20–59 years. It progressed sharply over 60 years. It is high in the patients between 70–79 years. Male HCM has been found to prevail over female HCM, i.e., 2.4:1 highly. The mean age is relatively stable, from 60.7 ± 12.9 years (2010) to 62.8 ± 13.2 (2016). Chest pain is the common clinical symptoms followed by Dyspnea. Hypertension is the general co-morbidity, and the proportion of heart failure increases from 15.3% to 38.5% in the years 2010 to 2016.

The prevailing study^19^ analyses the genetic associated variances in the expression as well as the outcome of the phenotypes that might present in numerous cardiac circumstances. It investigates the gender influences in HCM patients. The outcome of the analyses reveals that the 9427 patients are encompassed, and the female gender of almost 3719 is significantly related to the high risk of mortality (pooled OR = 1.63, 95% CI:1.26–2.10, *p* ≤ 0.001). The HCM associated mortality are pooled OR = 1.47, 95% CI:1.08–2.01, *p* = 0.015. The female gender are accompanied with the prognosis of HCM. The research study suggests that improvised care is required for the female gender, encompassing earlier detection and aggressive management. The gender-oriented analysis benefits the patients affected by the HCM.

### 2.1. Research gap

Even though conventional researchers have attempted to examine the genetics and gender differences that influence HCM prevalence, there exist possibilities for further advancements in the aspect of data collection. Hence, there is a need to focus on sample size and data congregation from a wider audience to gain ideal results. Thus, focusing on the diversified aspects can enhance the investigations regarding the HCM occurrences among the Indian populace. Therefore, the proposed research scrutinizes the comparison of the echocardiographic features with respect to subtypes of HCM. Further, the proposed study tends to reveal the impact of demographic features of genetics and gender on HCM patients.

## 3. Research methodology

### 3.1. Research design

The research design is denoted as the structure of the research method to finish the motive of the study^20^. The research design is envisioned to give an appropriate and suitable framework for the research. An essential judgment and resolution in the research design progression is the decision to be made concerning the research approach^21^. The process of providing a précised and complete framework on which the research will be processed is denoted as research design. In humble words, the research design is considered as the implementation of numerous procedures, processes and instruments to obtain data for research. The entire framework and the research flow of the present study are exposed in the research design. It integrates the appropriate way of approach for current research through answering questions. The research plan also encompasses a various combination of techniques and plans.

The quantitative method designates the occurrence through congregating numerical unchangeable data, which has been evaluated with the aid of mathematical approaches. This provides statistics associated questions of when, how, where, what, how much and how many. It incorporates the objective, logic, and number stance^22^. The research instrument utilized in the current research is a questionnaire, which aided in congregating data regarding the lean methodology and its optimization of the current study^23^. Quantitative research utilizes a survey as well as a questionnaire method for the gathering of primary data^24^.

The current study utilizes prospective and quantitative research methodology for congregating data. The research instrument utilized in this current study is evaluating the clinical features of the patients between the 1st March 2019 to 31st May 2021. It helps to capture the data regarding the interdisciplinary insights on the comparison of the echocardiographic characteristics in terms of genetics and gender of the patients. The survey is conducted among the patients of DMCH Ludhiana.

### 3.2. Study area

The research is accompanied by 103 patients, which aids in the fruitful achievement of the present research. The survey is conducted with the researcher’s support. The defendants who were surveyed for this research are extracted from the data of DMCH Ludhiana. This will enhance the significance of the study’s purpose, making the data gathering process easier.

### 3.3. Sample size and population

The sample size of the research study must be designated carefully to achieve an accurate outcome (Stratton, 2021). The appropriate sample participation will be selected to the extent of receiving the material concerning the clinical and demographical profile that has a significant impact on the prevalence of Hypertrophic cardiomyopathy among the patients of DMCH Ludhiana (Lakens, 2022). The present study applies purposive sampling. It selects a sample in accordance with the relevant understanding of the research determination. The convincing principles in the assortment of samples led to the in-depth outcome of the study. In research, the technique is considered the process of choosing a subset of individuals or units from a huge population to depict the entire population. The subgroup selection from the complete population is known as a sample, which is categorized into non-probability and probability sampling techniques. The probability sampling incorporates an aspect of random selection. It provides equal opportunities for the population to be chosen^25^. Few commonly employed probability techniques embrace cluster sampling, stratified sampling, random sampling, and systematic sampling, whereas the non-probability sampling technique incorporates diverse samples in a research outlook of subjects rather than random selection. The sampling method involves snowball sampling, purposive sampling, quota sampling, and self-selection sampling^26^. 103 participants are selected for the process of analysis. The targeted respondents are patients of DMCH Ludhiana where the data can be fetched for evaluating the clinical characteristics of Hypertrophic cardiomyopathy.

Inclusion Criteria: Any patient who had presented to DMCH/HDHI inpatient/outpatient clinic with an echocardiographic diagnosis of HCM.

Exclusion Criteria: The patients with Aortic valve stenosis, as well as the Physiological athlete’s heart, are incapable of the research purposes.

The sample size is calculated by the Cochran formulae,

 \begindispformula \text{Sample size}= \frac{{ \left( \text{Zscore} \right) }^{2}× \mathrm{S}.\mathrm{D}(1− \mathrm{S}.\mathrm{D})}{(\text{margin of error})^{2}} \enddispformula

The values of S.D   =   0.5, the margin of error is 0.096, and the Z score value is 1.96. Based on the above calculation, the sample size is 103.

### 3.4. Research instrument

The current study will accumulate data with the aid of Echocardiography. The enrolled patients are subjected to ECG, ECHO, BNP levels, Holter, and routine haemogram. The family members were screened for HCM with the support of ECG and Echocardiography. According to AHA (American Heart Association)/ACC (American College of Cardiology), the family members of the age group of 12 years and above are considered to be the first degree relatives of affected HCM patients.

The family screening strategies are as follows:

HCM, along with left ventricular hypertrophy, are detected with the aid of cardiovascular magnetic resonance and echocardiography.

Age (less than 12 years): They are treated as optional unless the following specifications:

1. There is premature death in the family due to HCM
2. Training program – competitive athlete
3. Prevailing the symptoms
4. Clinical suspicious of the initial stage left ventricular hypertrophy

Age (12–21 years): For every 12–18 months they are subjected to testing

Age (greater than 21 years): Imaging will be performed during the symptoms and at midlife or five-year intervals. The history of diagnosing late onset of HCM and malignant course are more prone to analysing frequently.

The genetic features and testing are elaborated as follows. The transmission of HCM in the form of Mendelian traits possesses an autosomal dominant pattern during the inheritance. It has been estimated that there is a fifty percent chance of the disease being inherited by the offspring encompassing the affected relative in their family. It is instigated through the mutation in eleven or more genes where the proteins are encoded with the thick as well as thin components of contractile myofilament present in the cardiac sarcomere and adjacent to the Z-disc. *β* Myosin heavy chain (MYH7) as well as myosin-binding protein C (MYBPC3) are the two main sarcomere genes that accounts for 70% of patients where the fourteen genotyping are successful. Alternatively, the supplementary genes, namely troponin I (TNNI3) and troponin T (TNNT2), are contributing five percent and below. The blood samples of five mL are gathered with the written consent mentioned in the study. The genetic testing is carried out by means of the Phenol-Chloroform technique to extract the genomic DNA. The Sanger method is suggested for the variant detection in the MYH7 gene for the sequence of exon 23, which is a hot spot region.

The criteria for the assessment of the Echocardiographs are as follows. It encompasses

1. LV Hypertrophy confirmation
2. Obstruction in the LVOT
3. SAM –Systolic Anterior Motion
4. LV functions in both systolic and diastolic assessment and size of left atrium

The 2D echocardiograph are utilized for the diagnosis of

1. The thickness of the maximal wall greater than 15 mm in the myocardial segment
2. The wall thickness ratio greater than 1.3 among the normotensive patients
3. The wall thickness ratio greater than 1.5 among the hypertensive patients

One-third of HCM patients possess an obstruction in the basal conditions. It is operated under the gradients ≥30 mm Hg. The second part of one-third will be labile as well as physiologically activated gradients, i.e., <30 mm Hg and ≥30 mm Hg in the rest and physiologic provocation, respectively. Finally, the remaining one-third will be the non-obstructive feature of HCM, i.e., at the <30 mm Hg at the rest and provocation. The conventional threshold will possess≥50 mm Hg at the marked gradients if the symptoms are not able to be optimized with the proper medications.

**Figure 1.**
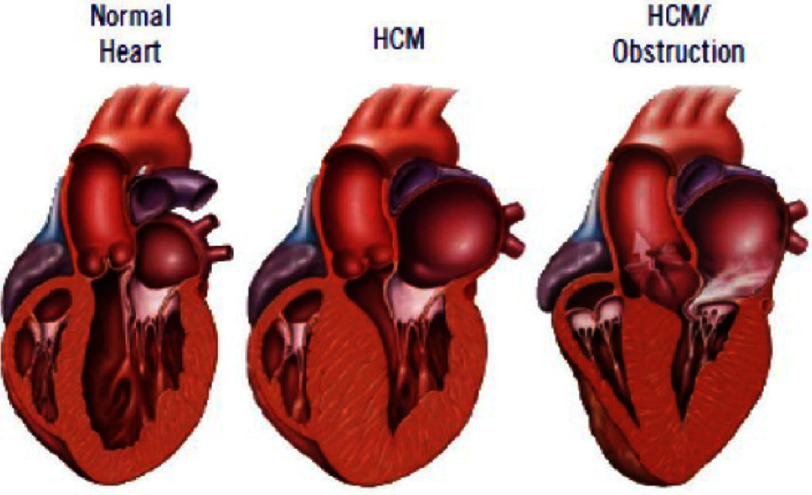
HCM subtypes^8^.

The diagnosis of HCM through electrocardiogram is as follows:

1. Enlargement of the left atrium
2. LVH associated with the abnormalities of ST segment or T-wave
3. Dagger-like Q waves in the leads of lateral>inferior
4. Inversions of T-wave in the apical HCM
5. WPW prevalence (Short PR and delta wave)
6. Dysrhythmias (Figure 1)

Hence, the primary endpoint of the research is the evaluation of echocardiographic characteristics in patients diagnosed with HCM. It focuses on key features such as the confirmation of LVH, obstruction in LVOT, SAM of the mitral valve, and the assessment of left ventricular function during both systolic and diastolic phases. These measurements are essential for understanding the clinical features and severity of HCM among the patient population at DMCH Ludhiana. The secondary endpoint encompasses genetic analysis and family screening for HC. Also, it includes identifying genetic mutations associated with the condition, particularly in critical genes such as MYH7 and MYBPC3. Additionally, family members of affected patients will be screened for early signs of HCM using ECG and Echocardiography, adhering to age-specific monitoring guidelines.

**Figure 2.**
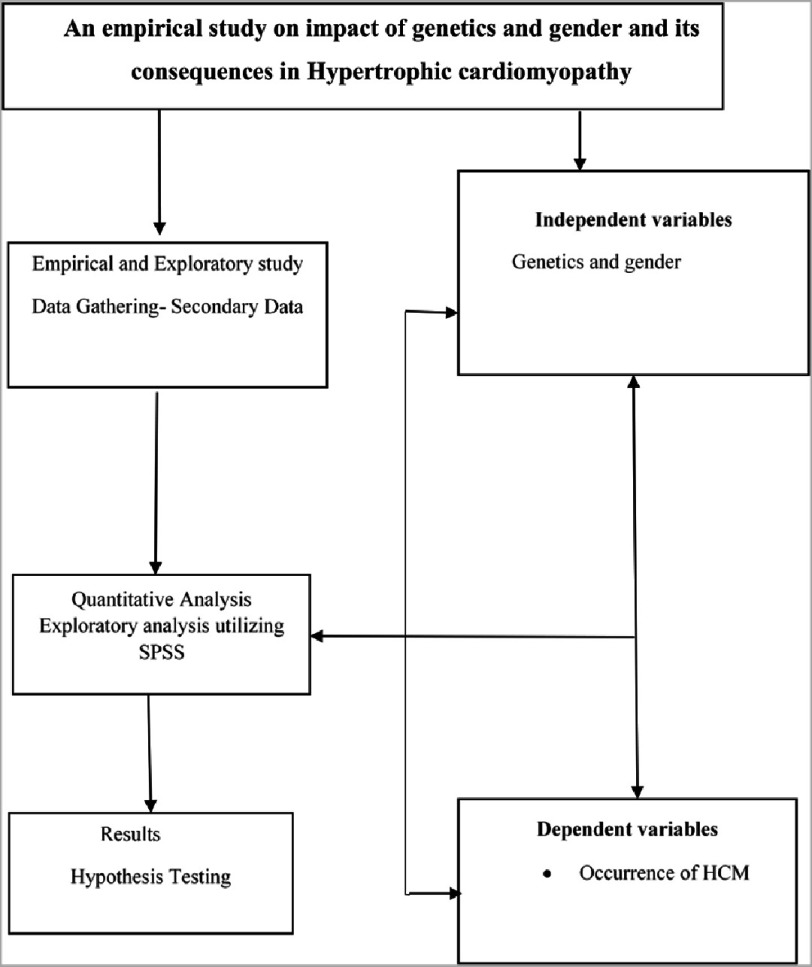
Research design.

### 3.5. Data analysis

Quantitative analysis is designated as a methodical phenomenon of assembling data and implementing computational, mathematical, and statistical approaches^27^. This technique is applied to gather data from respondents and define the outcomes for the targeted population^28^. The consequence of the method is resolute numerically. The numerical values are construed and also identify the upcoming research along with appropriate changes. It is multi-method in focus, naturalistic method and involved as an interpretative method to its subject matter^29^.

The quantitative data analysis method is used for the current study. The data are recorded using an Excel sheet to reveal study variables. The software tool is SPSS, and it is utilized for analysing the research variables in the Excel sheet. The outcome of the study is estimated by utilizing Chi-square and frequency.

The assumed techniques are employed to detect the data and validate the connotation amongst the variables of the present research. The outcome of the study constructs reveals the mode of interpretation and recommends further research. SPSS software aids in deriving the outcome of the present research and will be effective for documenting the constructs. Figure 2 illustrates the research framework of the present study. The tables and figures reveal the statistical results of the analysis. The tests are conducted to evaluate the hypothesis of the current research.

SPSS software is a set of software programs that analyses and studies the scientific information that is data relevant to research purposes and social science. This software provides the visual environment in a fast manner that covers both complex and smallest models. Surveys, market research, data mining, education institutions, industries, and other fields use the data collected from SPSS. It is popular software because of some common features like the user manual, which is well-documented, easy to understand the instruction language, and simplicity. The fundamental functionalities provided in SPSS are a statistical program intended for quantitative data analysis, which includes bivariate statistics, cross-tabulation and frequencies, text analysis, and modeler programming for the survey. Compared to other statistical tools, data analysis using SPSS requires less time, which results in fast outcomes.

The data analysis conducted is in the method of deep evaluation and statistical analysis. To generate appropriate results, the set of data is conducted resourcefully, and SPSS can also deal with a large set of data along with various formats. This finds the problem of the study and generates a solution in statistical form. Therefore, the present study will apply SPSS software to study and analyse the test hypothesis of the research. The chi-square test is a statistical technique that is utilized to examine the connection between categorical variables. It determines if the frequencies observed in a contingency table vary significantly from the expected frequencies based on the null hypothesis, which suggests that there is no relationship between the variables. It has been performed to analyze the clinical presentation, Echocardiographic findings, genetic analysis, and genotypes.

It was crucial to control for multiple comparisons to maintain the accuracy and dependability of the statistical results. To address this issue, the present research utilized the Bonferroni correction as a cautious measure against Type I errors. This adjustment involves dividing the original significance level (usually 0.05) by the total number of comparisons made in the study. The significance levels have been modified based on the number of tests performed and presented adjusted *p*-values alongside the original in the research findings. A finding is regarded as statistically significant when its *p*-value is below or equal to the specified level of significance (*p* ≤ 0.05). This indicates that there is an adequate condition to discard the null hypothesis in favor of the alternative hypothesis.

### Ethical consideration

All participants agreed to take part after receiving detailed information. This information covered the purpose of the research, the process of genetic testing, the possible risks and benefits, and what the results could mean for their health and family. Participants had the chance to ask any questions or raise any concerns they had. The consent forms were carefully crafted to make sure that they were clear and understandable, enabling participants to make informed choices about participating. Furthermore, participants were guaranteed that their information would be kept confidential and used only for research. They were also told that they could withdraw from the study at any point without affecting their medical treatment.

## 4. Results

The prospective study from the period of 1st March 2020 to 31st May 2021 encompassed 103 patients for research purposes. Among 103 patients, twenty-seven were admitted, and the remaining seventy-six were referred from an external clinic. Among 27 patients, nine were in follow-up, and the remaining eighteen were admitted for the first time. Among 76 patients, the twenty two were on follow up and the remaining fifty four patients are presented newly. Genetic analysis has been performed for 48 patients out of 103. The criteria for choosing 48 patients for genetic testing in the study were carefully developed to increase the chances of finding harmful mutations linked to hypertrophic cardiomyopathy. In order to better understand the genetic factors of HCM and its impact on patient care and family screening, the study concentrated on clinical symptoms, age, family background, and specific echocardiographic traits.

### Demographic analysis

### Age and gender

**Figure 3.**
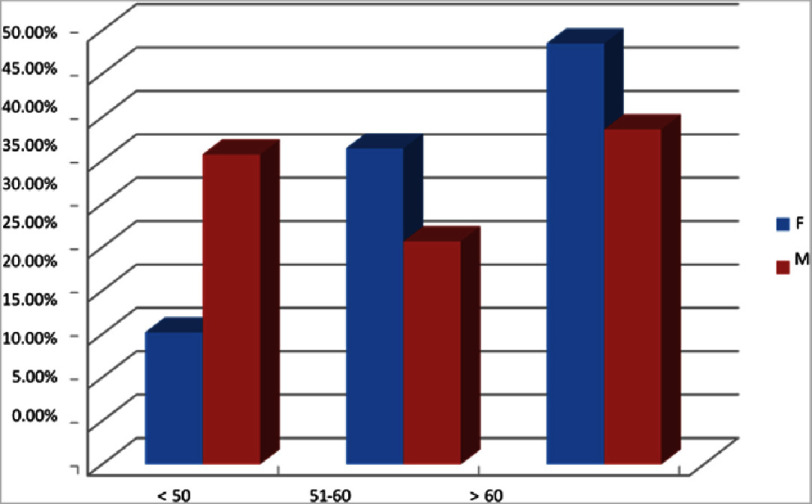
Age and gender-wise distribution. The demographic data shows that 29.1% of individuals under the age of 50 are male, with 35.7% male and 15.2% female. 29.1% of patients are aged between 51 and 60, with 25.7% male and 36.4% female. The average age of patients with HCM was found to be 56.28 ± 13.93 years. Secondly, gender differences were present: the average age for males was 54.67 ± 14.20 years, compared to 59.70 ± 12.87 years for females.

Table 1 reveals the age and gender wise distribution of the HCM patients. Among 103 HCM patients, 41.7% were lies in the age group of >60 years. 38.6% are males and 48.5% are females. Subsequently, 29.12% belongs to the age group <50 years whereas 35.7% are males and 15.2% are females. The remaining 29.12% are between the age group of 51–60 years, whereas 25.7% are males and 36.4% are females (Figure 3). The mean age of HCM patients is calculated and identifies that the minimum as well as maximum age was 17 and 90 years, respectively. The mean age was calculated as 56.28 ± 13.93 years. In terms of the gender wise, the mean age of males are 54.67 ± 14.20 years and female are 59.70 ± 12.87 years.

**Table 1:** Age and gender wise distribution of HCM patients.

**Parameters**		**Sex**				**Total**	**Chi-** **square value**	***p*-value**
		**F**		**M**				
Age group	<50	5	15.2%	25	35.7%	30	4.594	0.032
	51–60	12	36.4%	18	25.7%	30	1.232	0.267
	>60	16	48.5%	27	38.6%	43	0.906	0.341
Total		33	100.0%	70	100.0%	103		

### Clinical presentations

The gender wise impact on the HCM patients are calculated through the Chi-square analysis. The chief complaints are demonstrated in the following table.

### Chief complaints

From Table 2, the common chief complaint was SOB. Almost 80 patients (77.67%) are affected by the SOB, where 75.7% are males and 81.8% are females. Following this, 15.53% are affected by chest pain, where 14.3% are males and 18.2% are females. 14.56%, revealing that palpitation is the chief complaint (10% are males and 24.2% are females). 11.65% are affected by syncope, and the remaining 5.82% are affected by presyncope (5.7% are males and 6.1% are females) (Table 3).

**Table 2:** Gender wise distribution of HCM patients according to the chief complaints.

**Chief complaints**		**Sex**				**Total**	**Chi- square**	***p***
		**F**		**M**				
Chest pain	No	27	81.8%	60	85.7%	87	0.259	0.610
	Yes	6	18.2%	10	14.3%	16		
SOB	No	6	18.2%	17	24.3%	23	0.482	0.488
	Yes	27	81.8%	53	75.7%	80		
Palpitations	No	25	75.8%	63	90.0%	88	3.656	0.056
	Yes	8	24.2%	7	10.0%	15		
Syncope	No	28	84.8%	63	90.0%	91	0.578	0.447
	Yes	5	15.2%	7	10.0%	12		
Presyncope	No	31	93.9%	66	94.3%	97	0.005	0.944
	Yes	2	6.1%	4	5.7%	6		

**Table 3:** Medical history.

**Past history**		**Sex**				**Total**	***χ*2 value**	***p***
		**F**		**M**				
HTN	No	4	12.1%	25	35.7%	29	6.171	0.018
	Yes	29	87.9%	45	64.3%	74		
Bronchial Asthma	No	33	100.0%	70	100.0%	103		
Arrythmia	No	31	93.9%	66	94.3%	97	0.005	0.944
	Yes	2	6.1%	4	5.7%	6		
CVA	No	32	97.0%	69	98.6%	101	0.302	0.583
	Yes	1	3.0%	1	1.4%	2		
Diabetes mellitus	No	30	90.9%	69	98.6%	99	3.528	0.060
	Yes	3	9.1%	1	1.4%	4		
Heart failure	No	31	93.9%	70	100.0%	101	4.326	0.101
	Yes	2	6.1%	0	0.0%	2		
AICD implantation	No	33	100.0%	70	100.0%	103		

### Type of HCM

**Figure 4.**
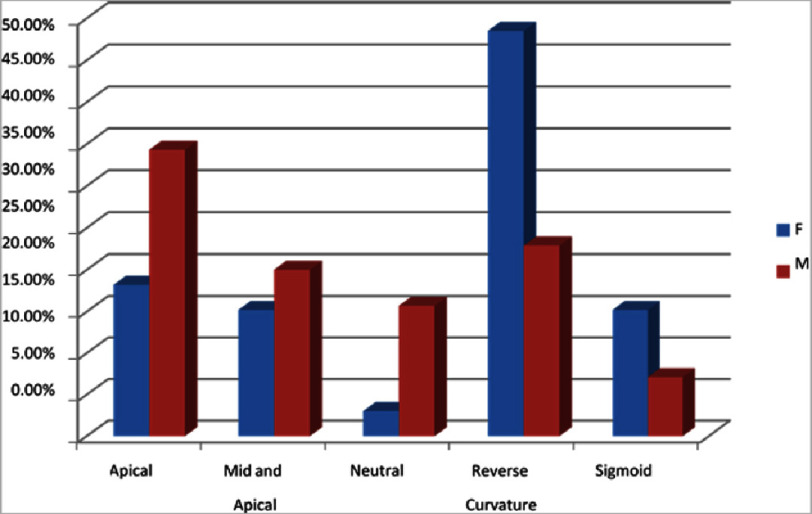
Type of HCM (gender wise distribution). Reverse curvature which contributes about 31.1% where 22.9% are males and 48.5% are females. 29.1% are apical (34.3% M and 18.2% F mid and apical in 19 (18.4%) patients (20% M and 15.2% F), neutral in 12 (11.7%) patients (15.7% M and 3% F), sigmoid in 10 (9.7%) patients (7.1% M and 15.2% F).

Figure 4 illustrates that the most common type is reverse curvature, which contributes about 31.1%, of which 22.9% are males and 48.5% are females. Following, 29.1% are apical (34.3% M and 18.2% F), mid and apical in 19 (18.4%) patients (20% M and 15.2% F), neutral in 12 (11.7%) patients (15.7% M and 3% F), and sigmoid in 10 (9.7%) patients (7.1% M and 15.2% F).

### Medical history

Among 103 patients, a history of HTN existed in 71.84% (*n* = 74) of patients, arrhythmia in 5.82% (*n* = 6), diabetes mellitus in 3.88% (*n* = 4), heart failure and CVA in 1.85% (*n* = 2). Comparing gender wise, past history of HTN was existing in 45 (64.3%) male and in 29 (87.9%) female patients. Past history of arrhythmia was found to be in 4 (5.7%) male and 2 (6.1%) female patients. The arrhythmia was found to be atrial fibrillation. Bronchial asthma, CVA, diabetes mellitus, heart failure, and AICD implantation were less prevalent.

### Personal history

Overall, out of a total of 103 patients, a personal history of OSA was found in 10.67% (*n* = 11) of patients, alcohol in 6.79% (*n* = 7), and smoking in 1.94% (*n* = 2). Comparing gender-wise, there were 2 (2.9%) male and none of the female patients who smoke. There were 7 (10%) male and none of the female patients who took alcohol. Personal history of OSA was in 6 (8.6%) of male and 5 (15.2%) of female patients. None of the male and female patients had a history of marathon running (Figure 4). Additionally, the family history of SCD are analysed and found to be 21.29% and comparing the gender wise impact, it has been found to be 20% are males and 27.3% are females.

### ECG findings

The patients were subjected to ECGs, and the gender-wise impacts analyzed.

**Figure 5.**
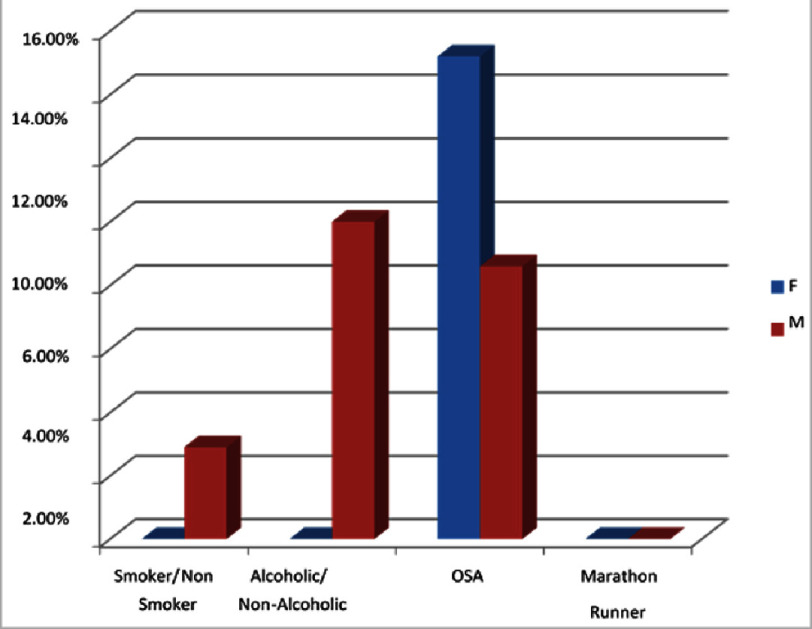
Personal history. OSA was found in 10.67% (*n* = 11) of patients, alcohol in 6.79% (*n* = 7), smoking in 1.94% (*n* = 2).

Table 4 demonstrates that among 103 patients, a normal ECG axis was found in 86.11% (*n* = 93) and left axis deviation in 9.7% (*n* = 10). Normal sinus rhythm (NSR) in 87.03% (*n* = 94), irregularly irregular in 6.79% (*n* = 7) and pacemaker rhythm in 1.85% (*n* = 2). LVH was found in 48.54% (*n* = 50). Prolonged QTc in 46.60% (*n* = 48). Giant T wave inversion in 48.54% (*n* = 50). Comparing gender-wise left axis deviation in ECG was found in 5 (7.1%) male and in 5 (15.2%) female patients where, as others, had normal axis. Irregularly irregular rhythm was found in 4 (5.7%) of male and 3 (9.1%) of female patients. LVH was found in 35 (50%) of male and 15 (45.5%) of female patients. Prolonged QTc was found in 27 (38.6%) male and 21 (63.6%) of female patients. Giant T wave inversion was found in 34 (48.6%) male and 16 (48.5%) female patients (Figure 5).

Table 5 compares gender wise mean PR interval in males and females was 158.18 ± 21.26 msec and 156 ± 24.86 msec. Mean QRS duration in males and females was 116.43 ± 6.82 msec and 119.70 ± 18.28 msec. Mean QTc (Bazett’s) in males and females was 461.87 ± 37.60 msec and 462.2 ± 26.99 msec.

**Table 4:** Gender wise distribution of HCM patients according to the ECG characteristics.

**ECG** **characteristics**		**Sex**				**Total**	***χ*2 value**	***p***
		**F**		**M**				
Axis	Left	5	15.2%	5	7.1%	10	1.641	0.200
	Normal (−30 to +100 degree)	28	84.8%	65	92.9%	93		
Rhythm	Irregularly irregular	3	9.1%	4	5.7%	7	4.838	0.089
	NSR	28	84.8%	66	94.3%	94		
	Pacemaker	2	6.1%	0	0.0%	2		
LVH (cornell voltage criteria)	No	18	54.5%	35	50.0%	53	2.227	0.328
	Yes	15	45.5%	35	50.0%	50		
QTc group	Normal (<460 msec in males and <470 msec in females)	12	36.4%	43	61.4%	55	5.662	0.021
	Prolonged (>460 msec in males and >470 msec in females)	21	63.6%	27	38.6%	48		
Giant T wave inversion (Negative T wave with voltage >10 mm)	No	17	51.5%	36	51.4%	53	0.000	0.993
	Yes	16	48.5%	34	48.6%	50		

**Table 5:** Gender-wise distribution of 12 lead ECG characteristics in HCM patients according to mean values.

**12 lead** **ECG characteristics**	**F**		**M**		***Z***	***p***
	**Mn**	**SD**	**Mn**	**SD**		
PR interval(ms)	156.00	24.86	158.18	21.26	−0.442	0.660
QRS duration(ms)	119.70	18.28	116.43	6.82	1.319	0.190
QTc (Bazett’s)(ms)	462.24	26.99	461.87	37.60	0.051	0.960

**Table 6:** Mean echocardiographic values.

**Echocardiographic** **parameters**	**F**		**M**		***Z***	***p***
	**Mn**	**SD**	**Mn**	**SD**		
Maximum wall thickness (mm)	25.52	4.87	24.33	3.48	1.414	0.161
Opposing wall ratio	2.43	1.07	1.93	0.89	2.167	0.013
LVID-S (mm)	26.12	5.17	30.06	6.19	−3.167	0.002
LVID-D (mm)	41.09	6.09	44.43	7.83	−2.157	0.033
LVPW-S(mm)	13.06	3.56	14.13	2.95	−1.596	0.114
LVPW-D (mm)	10.71	3.39	11.93	2.72	−1.953	0.054
IVS-S (mm)	24.09	6.69	20.76	6.26	2.467	0.015
IVS-D (mm)	21.58	6.16	18.39	5.90	2.533	0.013
LVOT gradient: resting (mm of Hg)	57.82	23.22	61.60	31.76	−0.356	0.725
Mitral valve- E/A	1.30	0.59	1.18	0.50	1.052	0.296
E/e’	18.26	5.91	15.24	5.31	2.603	0.011
IVRT	78.76	19.53	75.53	13.40	0.980	0.329
DT	183.58	49.71	177.59	45.14	0.608	0.544
PASP (mm of Hg)	33.52	10.49	30.41	9.13	1.533	0.128
LAVI(ml/m^2^ )	35.48	3.84	34.06	5.64	1.316	0.191
LAEF=(MaxV- MinV)/MaxV%	53.76	6.29	53.56	9.04	0.114	0.909
EFby2D echo	57.61	6.81	59.83	1.43	−2.625	0.010
EFby3D echo	57.70	6.51	59.86	1.90	−2.567	0.012
Strain derived EF	50.70	7.05	52.98	4.02	−2.085	0.040
Stroke volume(ml)	49.52	8.42	52.84	10.67	−1.574	0.119
LvMPI	0.52	0.07	0.50	0.08	1.152	0.252
GLS (- %)	14.71	4.05	14.15	3.85	0.667	0.506
GCS(- %)	16.45	4.04	16.80	4.87	−0.357	0.722
Longitudinal Lvstrain	
Apex	15.63	4.15	14.03	4.21	1.811	0.073
Mid	16.78	4.22	15.47	4.00	1.522	0.131
Base of lateral wall	16.55	4.57	15.96	4.46	0.621	0.536
The base of the septal wall	14.81	4.08	15.13	4.20	−0.362	0.718
LA strain(peak) %	19.80	2.76	21.06	4.02	−1.626	0.107

The echocardiographic characteristics are analyzed, and the distribution in terms of gender is calculated in the following table.

**Figure 6.**
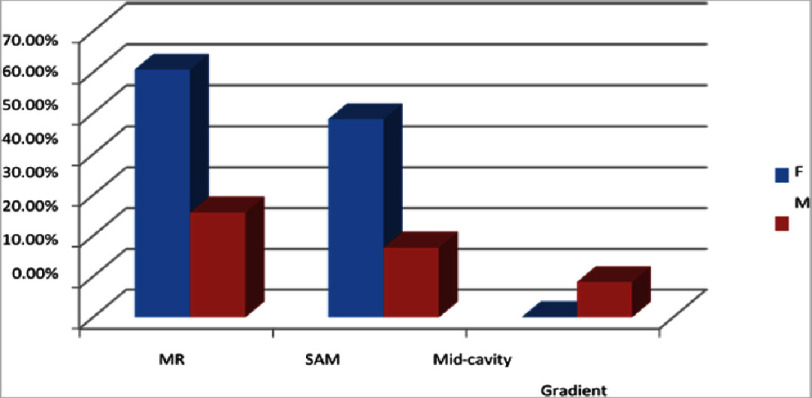
Echocardiographic characteristics. MR was found in 36.89% (*n* = 38) of patients, SAM in 27.13% (*n* = 28), and mid-cavity gradient in 5.82% (*n* = 6).

Figure 6 illustrates that among 103 patients, MR was found in 36.89% (*n* = 38) of patients, SAM in 27.13% (*n* = 28), and mid-cavity gradient in 5.82% (*n* = 6). Comparing gender wise, MR was found in 18 (25.7%) of male and 20 (60.6%) of male patients. SAM was found in 12 (17.1%) of male and 16 (48.5%) of female patients. Mid-cavity gradient was found in 6 (8.6%) of male and none of the female patients.

From Table 6, it has been identified that the mean opposing wall ratio was 1.93 ± 0.89 in male and 2.43 ± 1.07 in female patients. Mean LVID-S was 30.06 ± 6.19 mm in male and 26.12 ± 5.17 mm in female patients. Mean LVID-D was 44.43 ± 7.83 mm in male and 41.09  ± 6.09 mm in female patients. Mean IVS-S was 20.76 ± 6.26 mm in male and 24.09 ± 6.69 mm in female patients. Mean IVS-D was 18.39 ± 5.90 mm in male and 21.58 ± 6.16 mm in female patients. Mean E/e’ ratio was 15.24 ± 5.31 in male and 18.26  ± 5.91 in female patients. All the above parameters and mean EF of 2D, 3D, and strain derived Echocardiography showed statistical difference whereas all other parameters such as maximum wall thickness, LVPW-S and D, LVOT gradient resting and Valsalva, mitral valve E/A, E/e’, IVRT, DT, PASP, LAVI, LAEF, stroke volume, LvMPI, GLS, GCS, longitudinal Lv strain showed no statistical difference.

**Table 7:** Gender-wise distribution of HCM patients according to mean values of pulse rate and blood pressure.

**General physical** **examination**	**F**		**M**		***Z***	***p***
	**MN**	**SD**	**MN**	**SD**		
Pulse (/min)	78.03	14.19	78.94	12.30	−0.334	0.739
SBP (on medication)	131.03	17.23	130.54	15.84	0.142	0.888
DBP (on medication)	78.91	9.04	80.23	9.09	−0.689	0.493

### Pulse rate and blood pressure

The mean value of the pulse rate and blood pressure are estimated as follows

Table 7 compares the gender-wise mean pulse rates in males and females and is found to be 78.94 ± 12.30/min and 78.03 ± 14.19/min, respectively. Additionally, the mean SBP in males and females was 130.54 ± 15.84 mm of Hg and 131.03 ± 17.23 mm of Hg. Moreover, the mean DBP in males and females was 80.23 ± 9.09 mm of Hg and 78.91 ± 9.04 mm of Hg, respectively.

**Table 8:** Genetic analysis with types of HCM patients.

**Type**	**Genetic analysis (if required)**									**Total**	***χ*2**	***p***
	**CALR3**	**DTNA**	**MYBPC3**	**MYH6**	**MYH7**	**No** **abnormal gene found**	**Not done**	**PSEN2**	**TMEM43**			
Apical	0	0	4	0	1	6	19	0	0	30	35.042	0.326
Mid and Apical	0	0	2	0	1	7	9	0	0	19		
Neutral	0	0	1	0	1	2	7	1	0	12		
Reverse Curvature	1	1	2	0	6	7	14	0	1	32		
Sigmoid	0	0	1	1	2	0	6	0	0	10		
Total	1	1	10	1	11	22	55	1	1	103		

### Genetic analyses

The Table 8 illustrates the genetic analyses of the HCM patients. MYH7 gene mutation has been generally found in the reverse curvature. 6 out of 11 genes are positive (54.5%). In the case of MYBPC3, it is found in apical type. Four out of 10 genes are positive (40%).

### Genotype-phenotype correlations

Table 9 illustrates the gender wise distribution of the HCM patients as per the identified genes. Genetic analysis has been performed among 48 patients out of 103. 68.8% are male and 31.3% are female. No abnormal gene was found in the 22 patients (45.8%). The most common gene found was MYH7 in a total of 11 (22.19%) patients, i.e., in 15.2% M and 40% F. Second most common gene was found to be MYBPC3 in a total of 10 (20.83%) patients, i.e, in 21.2% M and 20% F. Less common genes were CALR3, DTNA, MYH6, PSEN2, and TMEM43.

**Table 9:** Gender-wise distribution of HCM patients according to the genes identified.

		**Sex**				**Total**	***χ*2**	***p***
		**F**		**M**				
Genetic analysis (if required)	CALR3	0	0.0%	1	3.0%	1	0.464	0.496
	DTNA	1	6.7%	0	0.0%	1	2.247	0.134
	MYBPC3	3	20.0%	7	21.2%	10	0.009	0.924
	MYH6	0	0.0%	1	3.0%	1	0.464	0.496
	MYH7	6	40.0%	5	15.2%	11	3.605	0.074
	No abnormal gene found	5	33.3%	17	51.5%	22	1.373	0.351
	PSEN2	0	0.0%	1	3.0%	1	0.464	0.496
	TMEM43	0	0.0%	1	3.0%	1	0.464	0.496
Total		15	100.0%	33	100.0%	48		

**Figure 7.**
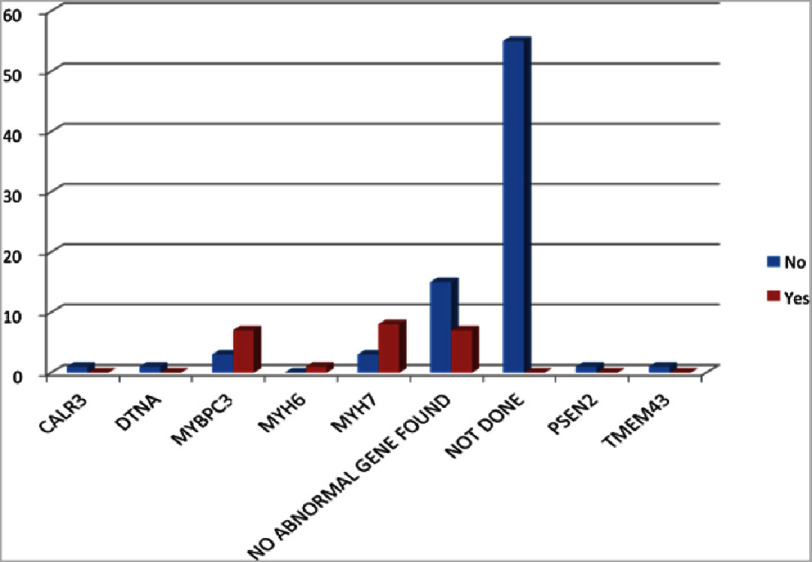
Family history. MYBPC3 gene was found in 30.4%, and the MYH7 gene was found in 34.8% of patients who had a family history of SCD.

The family history of SCD is analyzed and tabulated as follows. The implications of the analyses reveal that among 103 patients, a family history of sudden cardiac death was found in a total of 23 (22.3%) out of 103 patients. MYH7 gene was found in 34.8% (8 out of 23) of patients who had a family history of SCD. MYH7 gene was found in 34.8% (8 out of 23) of patients who had a family history of SCD. MYBPC3 gene was found in 30.4% (7 out of 23) of patients who had a family history of SCD. Another less common gene found was MYH6 in 4.3% (1 out of 23), and no abnormal gene was found in 30.4% (7 out of 23) of patients (Figure 7).

## 5. Discussion

The present research highlights that male predominance is higher compared to female, and the ratio was found to be 2.12:1. Similarly, the existing study^13^ evaluates the epidemiology and genetics of HCM. The sex ratio of males to females is found to be 3.7:1. Likewise, the prevailing studies^30^ and^31^ show similar implications.

The present study reveals that the mean age for the onset of HCM patients is during the sixth and seventh decades. This coincides with the prevailing studies of^17,18^. In contrast, the existing studies^8,14^ observe the mean age for the onset of HCM in the fourth decade.

The present research reveals that 57% of patients show a history of HTN. Similarly, the prevailing study^16^ evaluates the history of HTN at 34.3%. Moreover the predominance gene mutation is MYH7 and MYBPC3 and is prevailed in 44% of HCM patients. Likewise, the existing study^15^ shows similar implications of the gene mutations. Additionally, the predominance of the non-obstructive is found to be 74.76% compared to the obstructive (25.24%). Similarly, the existing study^14^ coincides with similar reports. The present research shows the mean posterior wall and IVS thickness observed to be 13.79 ± 3.18 and 21.83  ± 6.56 mm and is related to the prevailing studies of^14^. The present research reveals that the reverse curvature (31%) is the most prevalent type, and sigmoid (10%) is the least prevalent. Similarly, the prevailing study^17^ has the same reports. It also shows that the male and female distribution ratio is higher in apical, mid and apical, neutral variety and lower in reverse curvature and sigmoid type. It also reveals that shortness of breath is a common clinical presentation among HCM patients, which contributes to 77.67%, and similar implications were revealed in^30^. Likewise, contemporary studies^18^ evaluate that chest pain is the most clinical presentation.

## 5.1. Limitations

The current research has some constraints that could influence the outcome. Firstly, because this prospective observational study was only carried out in Ludhiana, limiting the patient pool to those with hypertrophic cardiomyopathy (HCM), the findings may not be representative of diverse populations. This geographical limitation could result in bias in selection, as the genetic and clinical traits of HCM can vary significantly between regions and demographics. Furthermore, bias may be introduced by the criteria for genetic testing, as not all patients underwent testing due to physician discretion or patient choice, which could skew the results and hinder drawing definitive conclusions about the prevalence of specific mutations in the wider HCM population. Additionally, potential influencing factors like age, other conditions, and environmental influences may not have been fully considered in our analysis, impacting genetic expression and clinical results. Lastly, the applicability of our findings is limited; although this research offers valuable insights into how genetic and gender factors affect HCM prevalence in our specific population, caution is necessary when applying these results to other groups or contexts. Despite these restrictions, this study provides significant knowledge about the genetic and gender-related aspects of HCM in India, which can guide clinical practice and future research efforts.

## 6. Conclusion

The current research offers valuable insights into the clinical and demographic characteristics of patients with HCM, emphasizing important findings regarding echocardiographic traits and the influence of gender and genetic factors on disease prevalence. The outcome shows a higher prevalence of HCM in men compared to women, with breathlessness being the main complaint in both sexes. The reverse curvature subtype is more common among males, while the apical type is frequently seen in females. Additionally, a substantial number of patients have a history of high blood pressure, and we noted notable differences in the size of the left ventricular cavity between men and women. Genetic testing revealed that mutations in the MYH7 and MYBPC3 genes are common, with specific mutations associated with a family history of sudden cardiac death. Moreover, non-obstructive HCM is more prevalent than obstructive forms. These findings have important implications for healthcare professionals treating HCM patients, as understanding the gender-specific symptoms and genetic basis can help tailor treatment strategies to improve patient outcomes. Future studies should focus on multi-center research involving diverse populations to improve the generalizability of results, explore long-term outcomes related to specific genetic mutations, and examine the effectiveness of targeted treatment plans based on gender and genetic markers. By addressing these areas, the present study can establish a more holistic approach to managing HCM and enhance the care provided to those affected.

## Conflict of Interest

The author reports that there is no conflict of interest.

## Declarations

### Funding

This research received no external funding.

## Acknowledgement

None.

## Data Availability

Data sharing is not applicable to this article as no datasets were generated.
